# Usefulness of plasma matrix metalloproteinase-9 levels in prediction of in-hospital mortality in patients who received emergent percutaneous coronary artery intervention following myocardial infarction

**DOI:** 10.18632/oncotarget.22401

**Published:** 2017-11-11

**Authors:** Jia-Jun Zhu, Qian Zhao, Hui-Juan Qu, Xiao-Mei Li, Qing-Jie Chen, Fen Liu, Bang-Dang Chen, Yi-Ning Yang

**Affiliations:** ^1^ Department of Cardiology, First Affiliated Hospital of Xinjiang Medical University, Urumqi, China; ^2^ Xinjiang Key Laboratory of Cardiovascular Disease Research, Urumqi, China; ^3^ Clinical Research Institute of Xinjiang Medical University, Urumqi, China

**Keywords:** MMP-9, STEMI, in-hospital mortality, coronary artery intervention

## Abstract

The aim of the present study was to investigate the predictive value of the plasma matrix metalloproteinase-9 (MMP-9) level at admission for in-hospital mortality in patients who received emergency percutaneous coronary intervention (PCI) following AMI. A single blood sample was collected at admission from 155 consecutive AMI patients who underwent emergent PCI. The plasma levels of MMP-9 value (528.9±191.6 ng/ml) were significantly higher in the patients who died (n=24) than in the survivors (385.4±236.0 ng/ml) during 14 days of hospitalization (*P*=0.005). The age, left ventricle wall motion score index (WMIS), Global Registry of Acute Coronary Events (GRACE) score and B-type natriuretic peptide (BNP) levels and GENSINI score at admission were significantly different between the patients who died and those who survived (*P*<0.001, *P*=0.004, *P*<0.001 and *P*<0.001, respectively). Cut-off concentrations for prediction of death was identified from receiver operator characteristic (ROC) curves. Using the cut-off value (MMP-9 level 398.2 ng/ml) to stratify the patients into two groups, the group with higher MMP-9 levels had a greater rate of in-hospital mortality than the lower level group (*P*<0.001). With the exception of the GRACE score, among all biomarkers measured, in stepwise multiple logistic regressions, only the MMP-9 level predicted the risk of in-hospital death after adjustment for all other risk factors (odds ratio 5.02, 95% CI 1.44 to 17.55). In conclusion, a higher MMP-9 level is an independent predictor of in-hospital death in AMI patients who received emergency PCI.

## INTRODUCTION

In the past two decades, China has experienced an epidemiological transition, ischemic heart disease kills more than 1 million people per year [1]. During the previous decade, the incidence of ST-segment elevation myocardial infarction (STEMI) in China has substantially increased. The quality of medical care for STEMI has improved; however, the in-hospital mortality in STEMI patients has not significantly changed [2]. Acute myocardial infarction (AMI) is categorized into two types, including STEMI and non-STEMI (NSTEMI). In China, The incidence of STEMI was far greater than that of NSTEMI [3]. So our study was not focus on the types of patients of AMI.

The identification of biomarkers that exhibit a predictive value is required for better risk stratification in AMI. There is solid evidence that the prognosis of AMI is associated with various factors, including dyslipidemia, smoking status, hypertension, diabetes mellitus, age, male gender, and plasma levels of B-type natriuretic peptide (BNP) and matrix metalloproteinase-9 (MMP-9) [4–7].

Among these biomarkers, MMP-9 is unique because it is closely associated with the development of atherosclerosis and instability of plaques [8–10]. Histological studies have indicated MMP-9 is expressed in atheroma lesions, and the MMP-9 expression was significantly increased in unstable plaques [11]. The levels of MMP-9, particularly in the coronary circulation [10, 12, 13], are significantly higher in acute coronary syndrome (ACS) patients than control subjects [14–18]. Moreover, MMP-9 plays a pivotal role in infarct healing, tissue repair and extracellular matrix (ECM) remodeling post-MI [19, 20].

Several investigators indicated that MMP-9 expressed within minutes of myocardial ischemia [21, 22], and may have a diagnostic values for ACS than hs-TnT (High sensitivity cardiac troponin T) at the earliest stage but not later stages [23]. Increasing evidence suggests that MMP-9can serve as a biomarker to evaluate the severity of coronary artery lesions and predict long-time of poor outcome and mortality of AMI [7, 24].

The aim of the present study was to investigate the predictive value of early MMP-9 levels for in-hospital mortality in STEMI patients following emergency percutaneous coronary intervention (PCI).

## RESULTS

**Figure 1:**
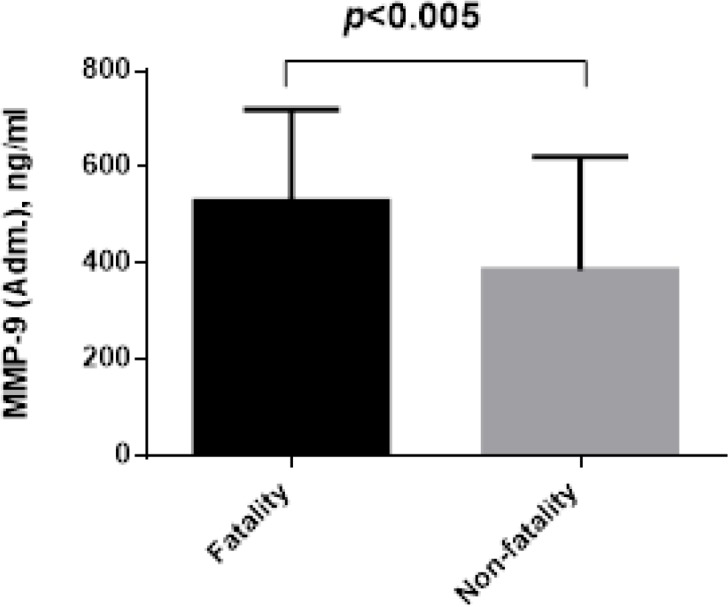
The difference admission plasma levels of MMP-9 between fatality and non-fatality

During 1-14 days, 24 (15.5%) patients died in the hospital. Comparison of the clinical characteristics between the fatality and non-fatality groups are presented in Table 1. The MMP-9 serum levels at admission was significantly increased in the patients who died compared with the survivors (528.9±191.6 ng/l vs. 385.4±236 ng/l, *P*=0.005, Figure 1). The patients who died were older and with complication (arrhythmia and cardiac shock) than the survivors and had significantly higher plasma BNP levels (*P*<0.0001), and Gensini scores (*P*=0.013), WMI (*P*<0.001), no-reflow (*P*<0.001), and also significantly different in GRACE score (*P*<0.001). There were no significant in differences in the other examined variables between the two groups.

**Table 1:** Comparison of characteristics and biomarkers at admission between the fatality and non-fatality groups

Variables	Total(n=155)	Fatality(n=24)	Non-fatality(n=131)	X^2^/t/z	*P* value
Age (years)	60±12	66±10	59±11	8.430	0.004
Male, n (%)	114 (73.5)	15 (62)	99 (75)	1.781	0.589
BMI	25.0±3.6	24.5±2.7	25.1±3.7	0.490	0.487
Hypertension, n (%)	76 (49.0)	12 (50.0)	64 (48.5)	0.011	0.918
Diabetes, n (%)	43 (27.7)	8 (33.3)	35 (26.7)	0.443	0.506
Current smoking, n (%)	89 (57.4)	15 (62.5)	74 (56.5)	0.300	0.584
MMP-9 (ng/ml)	407.7±235.4	528.9±191.6	385.4±236.0	2.806	0.005
MPV (fL)	10.4±1.2	10.5±1.4	10.4±1.2	0.571	0.571
Glucose (mmol/L)	10.2±5.1	10.9±3.9	9.2±4.0	1.738	0.080
Creatinine (mg/dL)	74.4(61.1∼90.7)	73.6(61.0∼86.8)	98.2(67.3∼125.1)	2.521	0.011
BNP (pmol/L)	507 (148∼1219)	5185 (682∼7963)	412 (30∼8663)	3.944	<0.001
Troponin I (mmol/L)	8.3 (1.8∼29.8)	14.4 (2.6∼25.3)	7.6 (0.8∼29.8)	0.623	0.533
CK AUC (IU/L)	57896(29710∼ 95342)	56537(33165∼92550)	69164(24502∼ 124218)	0.606	0.544
Peak CK (IU/L)	2484(1058∼4004)	2410(1139∼3929)	2910(455∼4203)	0.230	0.818
CKMB AUC (U/L)	4879(2561∼ 8519)	4833(2584∼7843)	7727(1615∼15637)	1.040	0.298
Peak CKMB (U/L)	216 (110∼385)	212(119∼342)	326(58∼689)	0.892	0.372
EF (%)	57±8	54±8	57±7	2.053	0.042
EF <50%, n (%)	23 (14.8)	6 (25.0)	17 (13.1)	2.267	0.132
WMIS	1.19±0.11	1.27±0.09	1.17±1.10	4.128	<0.001
GRACE score	120±26	146±26	115±22	6.249	<0.001
GENSINI score	61±34	83±43	58±32	2.510	0.013
arrhythmia (External temporary pacemaker)	25 (16.1)	10 (40.0)	15 (12.1)	9.991	0.002
Cardiac shock (IABP)	15 (10.0)	6 (25.0)	9 (8.0)	5.293	0.021
No-reflow	37 (23.8)	17 (70.8)	20 (15.8)	34.462	<0.001
**Medication, n (%)**					
Aspirin	154 (99.3)	24 (100)	130 (99.2)	0.183	0.669
β-blocker	101 (65.2)	16 (66.7)	89 (67.9)	0.015	0.902
ACEI or ARB	104 (67.1)	16 (66.7)	88 (67.2)	0.002	0.961
Statin	152 (98.0)	24 (100)	128 (97.7)	0.556	0.456

BMI, body mass index; MMP-9, matrix metalloproteinase-9; MPV, mean platelet volume; BNP, pro-B-type natriuretic peptide; EF, ejection function; IABP, intra-aortic balloon pump; ACE, angiotensin-converting enzyme; ARB, angiotensin receptor blocker; WMIS, left ventricle wall motion score index.

**Figure 2:**
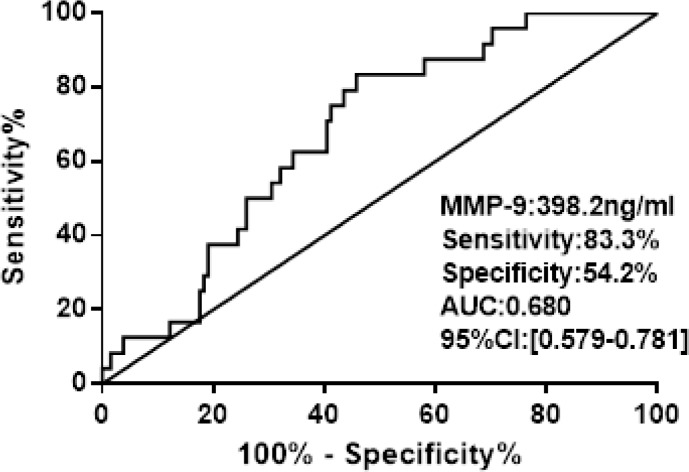
ROC curve analysis of admission MMP-9 for hospital death

The area under the ROC curve for prediction of the in-hospital mortality was 0.680 (95% CI 0.579–0.781;*P*< 0.001) for MMP-9, the cut-off value was 398.2 ng/l (Figure 2).

All 155 participants were further divided into high- and low-level groups, as Table 2, according to the MMP-9 cut-off value (398.2 ng/l). The two groups were subsequently compared in terms of age, male gender, current smoker, hypertension, diabetes mellitus, BNP level, body mass index (BMI), Troponin I level, glucose level, Creatinine, MPV, GRACE score (stratified into the following groups: Group 1: ≤ 108, low risk; Group 2: 109-140, moderate risk; and Group 3: >140, high risk), Gensini score, WMI, No-flow, fatality in hospital and impaired LV function (EF <50%). We identified greater in-hospital fatality (*P*=0.001), WMI (*P*=0.018) and no-flow (*P*=0.026) in the high-level group than the low-level group.

**Table 2:** Clinical characteristics of the study population according to cutoff of MMP-9

Variables	MMP-9<=398.2ng/ml (n=75)	MMP-9>398.2ng/ml (n=80)	X^2^/t/z	*P* value
Male, n (%)	51 (68.0)	60 (63.8)	2.299	0.129
Age (year)	58±11	61±11	1.362	0.175
BMI (kg/m2)	24.4±2.8	25.5±4.1	1.870	0.063
Diabetes mellitus, n (%)	21 (28.0)	22 (27.5)	0.004	0.945
Hypertension, n (%)	41 (54.6)	35 (43.8)	1.846	0.174
Current smoking, n (%)	39 (52.0)	50 (62.5)	1.746	0.186
MPV (fL)	10.5±1.3	10.3±1.1	0.904	0.367
Glucose (mmol/L)	8.8±3.5	10.1±4.5	1.806	0.073
Creatinine (mg/dL)	72.6 (64.6∼91.0)	75.5 (63.8∼86.8)	0.306	0.759
BNP (pmol/L)	532 (186∼1288)	447(146∼1046)	0.378	0.705
Troponin I (mmol/l)	8.1 (0.9∼30.4)	8.6 (1.8∼22.1)	0.019	0.985
GRACE score	117±23	122±27	1.208	0.228
Low-risk group, n (%)	30 (40.0)	27 (33.7)	1.999	0.368
Moderate -risk group, n (%)	33 (44.0)	33 (42.3)		
High-risk group, n (%)	12 (16.0)	20 (25.0)		
EF (%)	57±7	57±8	0.102	0.918
EF <50%, n (%)	10 (12.8)	13 (17.1)	0.556	0.456
WMIS	1.17±0.11	1.21±0.10	2.392	0.018
Gensini score	65±35	57±34	1.223	0.223
No-Reflow, n (%)	12 (16.0)	25 (31.3)	4.954	0.026
Fatality in hospital, n (%)	4 (5.3)	20 (25.0)	11.441	0.001

GRACE score groups: low-risk group≤108; moderate-risk group (109-140); and high-risk group >140;WMIS, left ventricle wall motion score index.

Next, the stepwise multivariable logistic regression method was used to adjust a range of risk factors, including male gender, age, BMI, diabetes mellitus, hypertension, current smoker, MPV, levels of MMP-9, glucose, BNP, troponin I, GRACE score, impaired LV function (left ventricular ejection function <50%), Gensini score, WMI, and no-flow, which were associated with in-hospital mortality. The results are indicated in Table 3. We determined that a high MMP-9 level (odds ratio 6.0, 95% confidence interval 1.79 to 20.13 *P*=0.004) and GRACE score (odds ratio 5.7, 95% confidence interval 2.59 to 12.76 *P*<0.001) were independent risk factors in the prediction of in-hospital mortality (Table 3).

**Table 3:** The association between risk factors and hospital death using stepwise multiple logistic regressions

Variable	Odds ratio	95% CI	*P* value
GRACE score group	5.7	2.588∼12.757	<0.001
MMP-9 adm. group(>398.2ng/ml)	6.0	1.787∼20.130	0.004

adm, administration.

Among 155 patients 24 (15.5%) patients died in the hospital. The shortest time to die after PCI is to die for Cardiogenic shock, after 5 hours. The cardiogenic shock is still the first to die in the first place (58.3%); malignant arrhythmia (29.2%) is the second cause of death, only three patients cause by cardiac rupture. Although MMP-9 serum levels was not statistically significant in the three categories of cause of death, the MMP-9 serum levels of three patients who died of cardiac rupture was significantly higher than other causes of death (Table 4).

**Table 4:** The cause of death and in the study population

Cause of death	N (%)	Time from admissionto death (hours)	MMP-9(ng/ml)
Cardiogenic shock	14 (58.3)	235 (31∼ 317)	496.8±195.1
Malignant arrhythmia	7 (29.2)	16 (11∼196)	501.0±159.3
Cardiac rupture	3 (12.5)	125 (73∼171)	744.2±139.4
t/z	-	5.200	2.430
*P* value	-	0.074	0.112

## DISCUSSION

During the 14 day study period, 15.5% of the AMI patients died in the hospital after PCI. All patients died as a result of cardiovascular events (cardiogenic shock, malignant arrhythmia and cardiac rupture). Compared with the survivors, the plasma levels of MMP-9 and BNP and the GRACE and GENSINI scores at admission were significantly higher in the group of patients who died in-hospital. Following an adjustment of all related clinical factors via multivariate logistic regression analysis, only MMP-9 and the GRACE score are independent factors in the prediction of in-hospital mortality in AMI patients after emergency PCI.

Compared various clinical risk factor for AMI, we found that Male gender, hypertension, diabetes mellitus, and current smoking/ex-smoking were not significantly different between the fatality and not-fatality groups. These results imply that these factors might not be major factors related to in-hospital fatality, in our patient population.

In our date, we found was no statistically significant reduction for the Troponin I levels between mortalities and survivors. Troponin I is recognized to be upregulated 3-4 hours after symptoms begin to worsen, with a peak at 11-24 hours [23, 25]. However, our study design was restricted to the symptoms at the time of a single blood sample, which was collected within 3-9 h after the onset of chest pain. Therefore, the peak Troponin I level may not be detected in this study.

As a gold standard to predict acute myocardial infarction patient prognosis, the GRACE score correlates with death in the hospital and at discharge based on large studies [26]. It is known that no matter in-hospital or out-hospital, cardiogenic shock, cardiac rupture or no-reflow is the common reasons death of patients after AMI [15]. So, our research focuses on these three causes of death.

The MMPs are expressed by macrophages, vascular smooth muscle cells, and endothelial cells in response to inflammatory stimuli and oxidative stress, all of which are involved in cardiovascular diseases [27]. MMPs include a large and an ever-expanding family, in which members share similar fundamental structural characteristics and are classified as endopeptidases with common functional domains and which are expressed in atherosclerotic plaques, induce degradation of extracellular matrix proteins [28, 29], including collagens and elastins, in atherosclerotic fibrous caps [30, 31]. MMP-9, known as gelatinase B or 92-kDa type IV collagenase, it can be released by neutrophils, macrophages, and fibroblasts [27], is expressed in atherosclerotic plaques, especially at the shoulder regions of those with thin fibrous caps, and plays a pathogenic role in the rupture or plaque vulnerability of atherosclerotic plaques [30, 32, 33]. Therefore our study was focus on the predicting value of MMP-9 levels for in hospital mortality in patient with AMI after PCI.

There are several potential explanations for this interesting association between elevated plasma MMP-9 levels and in-hospital mortality, including the following factors. First, Coronary atherosclerotic plaques are the pathological basis of coronary heart disease. AMI is usually initiated by rupture of atherosclerotic plaque, leading to intracoronary thrombosis and clinical sequelae. Human coronary atherectomy specimens revealed uniform and active synthesis of MMP-9 by macrophages and SMC in lesions from patients with unstable versus stable angina, found that MMP-9 is play a pathogenic role in the development of acute coronary ischemia [32], because it promoted the basement membrane rupture, resulting in smooth muscle cell migration and proliferation in the plaque, MMP - 9 at the same time promote the accumulation of mononuclear cells and macrophages [34], and these cells gradually turn into foam cells thus formed fatty nuclei of atherosclerotic plaques, moreover those precursor cells can secrete inducer and inflammation factors, it can stimulate activation of MMP-9 [34–36]. Those cells are the main source of MMP-9 in atherosclerotic plaques and can lead to the formation of [37] in new atherosclerotic plaques. The thrombosis is usually initiated by rupture of atherosclerotic plaque and exposure of highly thrombogenic plaque constituents to coronary blood flow. The extent of the resulting thrombus and its location in the coronary circulation contribute to the different resultant clinical syndromes [35, 36]. MMP-9 has been shown to be closely related to plaque rupture in most studies [11, 13, 16, 17, 37, 38]. It reduces the plaque fiber cap by degrading the fibrous cap matrix of atheromatous plaque, resulting in the formation and rupture of unstable plaques and induced myocardial infarction [8]. MMP-9 is closely related to ACS, although the current guidelines are not used to diagnose ACS, but there have been studies confirming that mmp-9 may be able to diagnose early ACS [23].

Second, a greater infarct size is associated with a higher the risk of mortality after AMI [39]. Our study showed that increases in value of MMP-9 levels were associated with increased WMIS, which is closely related to the area of acute myocardial infarction [40]. Experienced evidence shown that MMP-9-deficient mice exhibited a 35% smaller infarct size than wild type mice with a similar ischemic area. Attenuated activation of neutrophil infiltration and increased tissue inhibitor of metalloproteinase-1 (TIMP-1) levels accompanied this reduction in infarct size. This experimental result demonstrated established the important effect of MMP-9 on the infarct size [41]. Another experiment demonstrated that MMP-9-deficient mice exhibited attenuated ventricular dilatation and reduced collagen accumulation in response to left coronary occlusion [42, 43].

Third, in association with ECM degradation during LV remodeling, Left ventricular remodeling after myocardial infarction (MI) is a leading cause of congestive heart failure, and the degree of remodeling predicts morbidity and mortality [44, 45]. It is accepted that the cardiac extracellular matrix is critical for maintaining the structural integrity of the heart. Indeed, extracellular matrix degradation by MMP has been associated with slippage of myocyte fascicles and left ventricular wall thinning. accumulating evidence indicates that MMP-9 may be crucial in the process of ventricular remodeling [42, 43], as MMP-9-deficient mice have been determined to exhibit a reduced ventricular rupture rate following MI compared with wild type mice [41]. Selective MMP inhibition reduced ventricular dilatation and infarct wall thinning, and these results confirmed that collagenase activity was not a major determinant of the remodeling process [46]. Furthermore, MMP-9 has been identified to predict the outcome of ACS [7]. Moreover, excessive ECM degradation in the early phase of MI impairs infarct healing and early remodeling, consequently causes cardiac rupture [17]. Considering the evidence that NR (No-reflow) is the most common cause of in-hospital mortality, the pathophysiology of NR is complex and incompletely understood. Many phenomena contribute to NR, including leukocyte infiltration, vasoconstriction, activation of inflammatory pathways and cellular edema. Vascular damage and hemorrhage may also play important roles in the establishment of NR. M Dong, N Mu, F Ren, F Li, C Zhang and J Yang [43] determined that an elevated MMP-9 level in the culprit coronary artery may predict no-reflow in patients with AMI; ninety-five eligible AMI patients who underwent emergency PCI were consecutively recruited for this study, and the same results were obtained from univariate logistic analysis and multiple logistic regression analysis.

Based on the previously described findings, it may be postulated that there is likely a relationship between in-hospital fatality and plasma MMP-9 levels.

The single blood samples were obtained at the ER (Emergence Room) on arrival. Our study evaluated the ability of the plasma MMP-9 levels obtained from blood samples collected at admission to predict death in the hospital. A previous study confirmed that MMP-9 exhibited a higher diagnostic accuracy for ACS than hs-TnT at the earliest stage but not at later stages [23]. The results of this study indicated that MMP-9 not only serves as the earliest diagnostic marker of ACS [23] but also predicts in-hospital mortality in AMI patients after emergency PCI.

In contrast with several previous reports, our results did not indicate a relationship between the Troponin I levels and in-hospital mortality, and this discrepancy may be because our study design was restricted to the analysis of a single blood sample collected at admission to the ER.

## MATERIALS AND METHODS

### Patients and blood collection

This study consecutively recruited 155 AMI patients (mean age: 60±11 years and 114 men) who were admitted to the Cardiology Department of the First Affiliated Hospital of Xinjiang Medical University and who underwent emergency PCI from January 2012 to May 2014. The diagnosis of AMI was defined as a plasma CK-MB level >2-fold of the normal value or a cardiac troponin I (cTnI) >0.1 ng/ml after symptom onset together with at least one of the following: (1) chest pain that persisted for >20 min; or (2) diagnostic serial electrocardiograph (ECG) changes that consisted of new pathological Q waves or ST segment and T wave changes. The exclusion criteria included malignancy, renal replacement therapy, recent blood transfusion or surgery within one month. Demographic, clinical, biochemical and data were obtained. All patients received standard medical treatment, including oral a echocardiographic aspirin, clopidogrel 300 mg and emergency PCI, which conformed to the ESC guidelines [14]. The end point included the occurrence of all-cause mortality (Cardiogenic shock, Malignant arrhythmia, Cardiac rupture) in the hospital. We assessed the value of MMP-9 in the prediction of in-hospital mortality in AMI patients who underwent emergency PCI.

Blood samples were collected in the emergency room using tubes that contained EDTA. Blood was collected from all patients carefully and gently to avoid hemolysis before administration of any drugs at time of admission. The plasma was subsequently separated and stored at −80°C until ELISA. The mean symptom-to-sampling time was 6±2 hours.

GRACE score The GRACE risk prediction tool was previously described [47]. The score includes several variables (age, heart rate, systolic blood pressure, serum creatinine, congestive heart failure, in-hospital percutaneous coronary intervention or coronary aortic bypass grafting, history of MI, ST-segment depression, and elevated cardiac enzyme) was performed [48–50].

GENSINI score According to angiographic stenosis degree and scoring system designed by Gensini, 1 point is given for a stenosis of 0%–25%, 2 points for a stenosis of 25%–50%, 4 points for a stenosis of 50%–75%, 8 points for a stenosis of 75–90, 16 points for a stenosis of 90%–99% and 32 points for a narrowing of 100% [51].

Hypertension (defined as systolic blood pressure >140 mm Hg and diastolic blood pressure >90mm Hg in more than one measurement or being under antihypertensive drug treatment).

Transthoracic echocardiography was performed at admission to determine left ventricle wall motion index score (WMIS) that was measured using a standard 16-segment model from parasternal long and short axes, and apical two- and four-chamber views. Each LV segment is scored as 0 – hyperkinetic, 1 – normal, 2 – hypokinetic, 3 – akinetic, and 4 – dyskinetic. The total divided by the number of segments analyzed gives an overall score, higher values indicating more impaired LV function.(Vivid 7 or E9, GE Medical Systems, USA).

No-reflow (NR) was diagnostic from Thrombolysis in Myocardial Infarction (TIMI) scale, the absence of reflow is classified according to the TIMI angiographic scale as a grade<2 in the culprit coronary artery after the PCI procedure [52].

### Laboratory tests

ELISA for MMP-9: Briefly, MMP-9 antibodies (200 ng/100 μl, R&D Systems, USA) were coated onto 96-well plates overnight at room temperature (20°C). The plates were subsequently blocked using 10% (v/v) fetal bovine serum. Plasma samples and standards were added and incubated overnight. After washing, the tracer antibody (5 ng/100 μl biotinylated goat antibody specific for MMP-9) was added and incubated for 2 h. After washing, the MMP-9 levels were determined using MAE-labeled streptavidin according to the manufacturer's instructions.

Other biochemical assays: The results for the mean platelet volume (MPV), glucose level, creatinine, a component of BNP, Troponin I level, and these factors originated from the First Affiliated Hospital of Xinjiang Medical University central laboratory. The remaining BNP was derived from point-of-care testing (Triage® meter plus, Biosite® Incorporated, USA).

### Statistical analysis

Statistical analyses were performed using SPSS Version 22. All numeric variables with a Gaussian distribution are presented as the means±SD, whereas variables with a non-Gaussian distribution are presented as medians and interquartile ranges. Categorical variables are presented as numbers and percentages. The baseline characteristics were analyzed using the Chi-squared test, Student's *t* test or the Mann-Whitney-Wilcoxon test. A stepwise multivariable logistic regression was used to analyze all potential risk factors associated with in-hospital mortality, as well as overall mortality. A *P* value <0.05 indicated a statistic significance association.

We estimated the strength of association with outcome using individual plasma serum of MMP-9 and in-hospital death identified from receiver operator characteristic (ROC) curves (Figure 1). These cut-off values were identified as the point on the curve showing the highest combination of sensitivity and specificity.

## References

[1] Govender R, De Greef J, Delport R, Becker PJ, Vermaak WJ (2008). Biological variation of ischaemia-modified albumin in healthy subjects. Cardiovasc J Afr.

[2] Li J, Li X, Wang Q, Hu S, Wang Y, Masoudi FA, Spertus JA, Krumholz HM, Jiang L, China PC (2015). ST-segment elevation myocardial infarction in China from 2001 to 2011 (the China PEACE-Retrospective Acute Myocardial Infarction Study): a retrospective analysis of hospital data. Lancet.

[3] Gao R, Patel A, Gao W, Hu D, Huang D, Kong L, Qi W, Wu Y, Yang Y, Harris P, Algert C, Groenestein P, Turnbull F, CPACS Investigators (2008). Prospective observational study of acute coronary syndromes in China: practice patterns and outcomes. Heart.

[4] Abbott RD, Donahue RP, Kannel WB, Wilson PW (1988). The impact of diabetes on survival following myocardial infarction in men vs women. The Framingham Study. JAMA.

[5] Butler WJ, Ostrander LD, Carman WJ, Lamphiear DE (1985). Mortality from coronary heart disease in the Tecumseh study. Long-term effect of diabetes mellitus, glucose tolerance and other risk factors. Am J Epidemiol.

[6] Arakawa N, Nakamura M, Aoki H, Hiramori K (1996). Plasma brain natriuretic peptide concentrations predict survival after acute myocardial infarction. J Am Coll Cardiol.

[7] Kelly D, Khan SQ, Thompson M, Cockerill G, Ng LL, Samani N, Squire IB (2008). Plasma tissue inhibitor of metalloproteinase-1 and matrix metalloproteinase-9: novel indicators of left ventricular remodelling and prognosis after acute myocardial infarction. Eur Heart J.

[8] Zayani Y, Allal-Elasmi M, Jacob MP, Zidi W, Zaroui A, Feki M, Mourali S, Mechmech R, Kaabachi N (2013). Peripheral blood levels of matrix and inflammatory mediators are elevated in Tunisian patients with acute coronary syndromes. Clin Lab.

[9] Guzel S, Serin O, Guzel EC, Buyuk B, Yilmaz G, Guvenen G (2013). Interleukin-33, matrix metalloproteinase-9, and tissue inhibitor [corrected] of matrix metalloproteinase-1 in myocardial infarction. Korean J Intern Med.

[10] Johnson C, Galis ZS (2004). Matrix metalloproteinase-2 and -9 differentially regulate smooth muscle cell migration and cell-mediated collagen organization. Arterioscler Thromb Vasc Biol.

[11] Fukuda D, Shimada K, Tanaka A, Kusuyama T, Yamashita H, Ehara S, Nakamura Y, Kawarabayashi T, Iida H, Yoshiyama M, Yoshikawa J (2006). Comparison of levels of serum matrix metalloproteinase-9 in patients with acute myocardial infarction versus unstable angina pectoris versus stable angina pectoris. Am J Cardiol.

[12] Inokubo Y, Hanada H, Ishizaka H, Fukushi T, Kamada T, Okumura K (2001). Plasma levels of matrix metalloproteinase-9 and tissue inhibitor of metalloproteinase-1 are increased in the coronary circulation in patients with acute coronary syndrome. Am Heart J.

[13] Bittner A, Alcaino H, Castro PF, Perez O, Corbalan R, Troncoso R, Chiong M, Mellado R, Moraga F, Zanolli D, Winter JL, Zamorano JJ, Díaz-Araya G, Lavandero S (2010). Matrix metalloproteinase-9 activity is associated to oxidative stress in patients with acute coronary syndrome. Int J Cardiol.

[14] Steg PG, James SK, Atar D, Badano LP, Blömstrom-Lundqvist C, Borger MA, Di Mario C, Dickstein K, Ducrocq G, Fernandez-Aviles F, Gershlick AH, Giannuzzi P, Halvorsen S (2012). Task Force on the management of ST-segment elevation acute myocardial infarction of the European Society of Cardiology (ESC). ESC Guidelines for the management of acute myocardial infarction in patients presenting with ST-segment elevation. Eur Heart J.

[15] Song L, Yang YJ, Lu SZ, Yang XC, Li HW, Guo JC, Gao W, Huang CL, Fang Q, Wu MY, Hao HJ (2012). [Cause of in-hospital death among acute myocardial infarction patients undergoing primary percutaneous coronary intervention in Beijing]. [Article in Chinese]. Zhonghua Xin Xue Guan Bing Za Zhi.

[16] Romanic AM, Harrison SM, Bao W, Burns-Kurtis CL, Pickering S, Gu J, Grau E, Mao J, Sathe GM, Ohlstein EH, Yue TL (2002). Myocardial protection from ischemia/reperfusion injury by targeted deletion of matrix metalloproteinase-9. Cardiovasc Res.

[17] Heymans S, Luttun A, Nuyens D, Theilmeier G, Creemers E, Moons L, Dyspersin GD, Cleutjens JP, Shipley M, Angellilo A, Levi M, Nübe O, Baker A (1999). Inhibition of plasminogen activators or matrix metalloproteinases prevents cardiac rupture but impairs therapeutic angiogenesis and causes cardiac failure. Nat Med.

[18] Ducharme A, Frantz S, Aikawa M, Rabkin E, Lindsey M, Rohde LE, Schoen FJ, Kelly RA, Werb Z, Libby P, Lee RT (2000). Targeted deletion of matrix metalloproteinase-9 attenuates left ventricular enlargement and collagen accumulation after experimental myocardial infarction. J Clin Invest.

[19] Brauer PR (2006). MMPs--role in cardiovascular development and disease. Front Biosci.

[20] Galis ZS, Khatri JJ (2002). Matrix metalloproteinases in vascular remodeling and atherogenesis: the good, the bad, and the ugly. Circ Res.

[21] Etoh T, Joffs C, Deschamps AM, Davis J, Dowdy K, Hendrick J, Baicu S, Mukherjee R, Manhaini M, Spinale FG (2001). Myocardial and interstitial matrix metalloproteinase activity after acute myocardial infarction in pigs. Am J Physiol Heart Circ Physiol.

[22] Lu L, Gunja-Smith Z, Woessner JF, Ursell PC, Nissen T, Galardy RE, Xu Y, Zhu P, Schwartz GG (2000). Matrix metalloproteinases and collagen ultrastructure in moderate myocardial ischemia and reperfusion *in vivo*. Am J Physiol Heart Circ Physiol.

[23] Kobayashi N, Hata N, Kume N, Yokoyama S, Shinada T, Tomita K, Kitamura M, Shirakabe A, Inami T, Yamamoto M, Seino Y, Mizuno K (2011). Matrix metalloproteinase-9 for the earliest stage acute coronary syndrome. Circ J.

[24] Wang KF, Huang PH, Chiang CH, Hsu CY, Leu HB, Chen JW, Lin SJ (2013). Usefulness of plasma matrix metalloproteinase-9 level in predicting future coronary revascularization in patients after acute myocardial infarction. Coron Artery Dis.

[25] Keller T, Zeller T, Peetz D, Tzikas S, Roth A, Czyz E, Bickel C, Baldus S, Warnholtz A, Frohlich M, Sinning CR, Eleftheriadis MS, Wild PS (2009). Sensitive troponin I assay in early diagnosis of acute myocardial infarction. N Engl J Med.

[26] Investigators. GRACE (2001). Rationale and design of the GRACE (Global Registry of Acute Coronary Events) Project: a multinational registry of patients hospitalized with acute coronary syndromes. Am Heart J.

[27] Newby AC (2008). Metalloproteinase expression in monocytes and macrophages and its relationship to atherosclerotic plaque instability. Arterioscler Thromb Vasc Biol.

[28] Birkedal-Hansen H (1995). Proteolytic remodeling of extracellular matrix. Curr Opin Cell Biol.

[29] Dollery CM, McEwan JR, Henney AM (1995). Matrix metalloproteinases and cardiovascular disease. Circ Res.

[30] Galis ZS, Sukhova GK, Lark MW, Libby P (1994). Increased expression of matrix metalloproteinases and matrix degrading activity in vulnerable regions of human atherosclerotic plaques. J Clin Invest.

[31] Shah PK, Falk E, Badimon JJ, Fernandez-Ortiz A, Mailhac A, Villareal-Levy G, Fallon JT, Regnstrom J, Fuster V (1995). Human monocyte-derived macrophages induce collagen breakdown in fibrous caps of atherosclerotic plaques. Potential role of matrix-degrading metalloproteinases and implications for plaque rupture. Circulation.

[32] Brown DL, Hibbs MS, Kearney M, Loushin C, Isner JM (1995). Identification of 92-kD gelatinase in human coronary atherosclerotic lesions. Association of active enzyme synthesis with unstable angina. Circulation.

[33] Ishino S, Mukai T, Kume N, Asano D, Ogawa M, Kuge Y, Minami M, Kita T, Shiomi M, Saji H (2007). Lectin-like oxidized LDL receptor-1 (LOX-1) expression is associated with atherosclerotic plaque instability--analysis in hypercholesterolemic rabbits. Atherosclerosis.

[34] Luttun A, Lutgens E, Manderveld A, Maris K, Collen D, Carmeliet P, Moons L (2004). Loss of matrix metalloproteinase-9 or matrix metalloproteinase-12 protects apolipoprotein E-deficient mice against atherosclerotic media destruction but differentially affects plaque growth. Circulation.

[35] Falk E (1983). Plaque rupture with severe pre-existing stenosis precipitating coronary thrombosis. Characteristics of coronary atherosclerotic plaques underlying fatal occlusive thrombi. Br Heart J.

[36] Davies MJ, Thomas AC (1985). Plaque fissuring--the cause of acute myocardial infarction, sudden ischaemic death, and crescendo angina. Br Heart J.

[37] Kai H, Ikeda H, Yasukawa H, Kai M, Seki Y, Kuwahara F, Ueno T, Sugi K, Imaizumi T (1998). Peripheral blood levels of matrix metalloproteases-2 and -9 are elevated in patients with acute coronary syndromes. J Am Coll Cardiol.

[38] Park HJ, Baek JY, Shin WS, Kim DB, Jang SW, Shin DI, Koh YS, Seo SM, Uhm JS, Kim TH, Kim PJ, Chang K, Chung WS (2011). Soluble receptor of advanced glycated endproducts is associated with plaque vulnerability in patients with acute myocardial infarction. Circ J.

[39] Burns RJ, Gibbons RJ, Yi Q, Roberts RS, Miller TD, Schaer GL, Anderson JL, Yusuf S, CORE Study Investigators (2002). The relationships of left ventricular ejection fraction, end-systolic volume index and infarct size to six-month mortality after hospital discharge following myocardial infarction treated by thrombolysis. J Am Coll Cardiol.

[40] Zhang Y, Takagawa J, Sievers RE, Khan MF, Viswanathan MN, Springer ML, Foster E, Yeghiazarians Y (2007). Validation of the wall motion score and myocardial performance indexes as novel techniques to assess cardiac function in mice after myocardial infarction. Am J Physiol Heart Circ Physiol.

[41] Sundstrom J, Evans JC, Benjamin EJ, Levy D, Larson MG, Sawyer DB, Siwik DA, Colucci WS, Sutherland P, Wilson PW, Vasan RS (2004). Relations of plasma matrix metalloproteinase-9 to clinical cardiovascular risk factors and echocardiographic left ventricular measures: the Framingham Heart Study. Circulation.

[42] Tao ZY, Cavasin MA, Yang F, Liu YH, Yang XP (2004). Temporal changes in matrix metalloproteinase expression and inflammatory response associated with cardiac rupture after myocardial infarction in mice. Life Sci.

[43] Dong M, Mu N, Ren F, Li F, Zhang C, Yang J (2015). Matrix metalloproteinase-9 in the culprit coronary artery and myocardial no-reflow. Am J Med Sci.

[44] White HD, Braunwald E (1998). Applying the open artery theory: use of predictive survival markers. Eur Heart J.

[45] Pfeffer MA, Braunwald E (1990). Ventricular remodeling after myocardial infarction. Experimental observations and clinical implications. Circulation.

[46] Lindsey ML, Gannon J, Aikawa M, Schoen FJ, Rabkin E, Lopresti-Morrow L, Crawford J, Black S, Libby P, Mitchell PG, Lee RT (2002). Selective matrix metalloproteinase inhibition reduces left ventricular remodeling but does not inhibit angiogenesis after myocardial infarction. Circulation.

[47] Fox KA, Dabbous OH, Goldberg RJ, Pieper KS, Eagle KA, Van de Werf F, Avezum A, Goodman SG, Flather MD, Anderson FA, Granger CB (2006). Prediction of risk of death and myocardial infarction in the six months after presentation with acute coronary syndrome: prospective multinational observational study (GRACE). BMJ.

[48] Tang EW, Wong CK, Herbison P (2007). Global Registry of Acute Coronary Events (GRACE) hospital discharge risk score accurately predicts long-term mortality post acute coronary syndrome. Am Heart J.

[49] Ramsay G, Podogrodzka M, McClure C, Fox KA (2007). Risk prediction in patients presenting with suspected cardiac pain: the GRACE and TIMI risk scores versus clinical evaluation. QJM.

[50] Eagle KA, Lim MJ, Dabbous OH, Pieper KS, Goldberg RJ, Van de Werf F, Goodman SG, Granger CB, Steg PG, Gore JM, Budaj A, Avezum A, Flather MD (2004). A validated prediction model for all forms of acute coronary syndrome: estimating the risk of 6-month postdischarge death in an international registry. JAMA.

[51] Emond M, Mock MB, Davis KB, Fisher LD, Holmes DR, Chaitman BR, Kaiser GC, Alderman E, Killip T (1994). Long-term survival of medically treated patients in the Coronary Artery Surgery Study (CASS) Registry. Circulation.

[52] TIMI Study Group (1985). The thrombolysis in myocardial infarction (TIMI) trial. Phase I findings. N Engl J Med.

